# Implications of sex-specific selection for the genetic basis of disease

**DOI:** 10.1111/eva.12097

**Published:** 2013-09-04

**Authors:** Edward H Morrow, Tim Connallon

**Affiliations:** 1 Evolution, Behaviour and Environment Group, School of Life Sciences, University of SussexBrighton, UK; 2 Department of Molecular Biology and Genetics, Cornell UniversityIthaca, NY, USA

**Keywords:** contemporary evolution, ecological genetics, evolutionary medicine, evolutionary theory, sexual selection

## Abstract

Mutation and selection are thought to shape the underlying genetic basis of many common human diseases. However, both processes depend on the context in which they occur, such as environment, genetic background, or sex. Sex has widely known effects on phenotypic expression of genotype, but an analysis of how it influences the evolutionary dynamics of disease-causing variants has not yet been explored. We develop a simple population genetic model of disease susceptibility and evaluate it using a biologically plausible empirically based distribution of fitness effects among contributing mutations. The model predicts that alleles under sex-differential selection, including sexually antagonistic alleles, will disproportionately contribute to genetic variation for disease predisposition, thereby generating substantial sexual dimorphism in the genetic architecture of complex (polygenic) diseases. This is because such alleles evolve into higher population frequencies for a given effect size, relative to alleles experiencing equally strong purifying selection in both sexes. Our results provide a theoretical justification for expecting a sexually dimorphic genetic basis for variation in complex traits such as disease. Moreover, they suggest that such dimorphism is interesting – not merely something to control for – because it reflects the action of natural selection in molding the evolution of common disease phenotypes.

## Introduction

The science of Darwinian medicine seeks to provide ultimate explanations for the existence of the diseases and conditions that afflict modern humans (Nesse and Williams 1996). Its success relies on the principle that we, like every other living organism, are a product of evolutionary processes. To date, investigators studying the genetics of human disease often assume that disease-predisposing alleles are deleterious (Guttmacher and Collins 2002; Bodmer and Bonilla 2008) and that a process of recurrent mutation might explain their presence within the population (Fisher 1958). But not all mutations are universally deleterious and the context (both environmental and genetic) in which genes are expressed can be important (see Ober and Vercelli 2011; Huff et al. 2012). Recent work in evolutionary and quantitative genetics has shown that mutations often have distinct effects in males and females, by differentially affecting male and female traits (e.g., Mackay 2001) or by having sex-specific fitness consequences (e.g., Rice and Chippindale 2001; Whitlock and Agrawal 2009; Innocenti et al. 2011). Mutations harmful to both sexes may nevertheless have different severities in each sex. Furthermore, mutations harmful to one sex can be beneficial to the other – that is, their effects may be ‘sexually antagonistic’ (Rice 1992). However, despite the clear evidence of sex-specific genetic effects on the phenotype, the effects of sex-specific evolutionary processes on the genetic basis of disease have not been extensively explored.

There are two reasons why we might expect sex-specific selection to play an important role in the genetic basis of human disease. First, association studies in humans and QTL analyses from model organisms seem particularly adept at identifying genetic variants that have more severe effects on one sex than the other (Ober et al. 2008). These findings lend plausibility to the argument that the genetic basis of disease differs considerably between males and females, that is, that disease phenotypes have sex-specific genetic architectures. Second, population genetics theory predicts that mutations with asymmetric or opposing effects on male and female fitness can reach higher population frequencies than mutations under similar patterns of selection in both sexes (e.g., Haldane 1937, 1962; Kidwell et al. 1977; Hansen and Price 1999; Morrow et al. 2008; Whitlock and Agrawal 2009). Such alleles may disproportionately contribute to overall genetic variability, even when most mutations have similar effects in males and females.

While sex-specific selection should be relevant to complex (polygenic) disease, particularly to the extent that the manifestation of disease decreases fitness, we currently lack a quantitative theoretical framework to address the likely importance of sex-specific processes for disease genetics and to compare directly the relative contributions to phenotypic variance of alleles with symmetric versus asymmetric fitness effects in the two sexes. Using a simple population genetic model, we show that a sexually dimorphic genetic architecture for fitness- and disease-related phenotypes readily emerges when mutational effects are imperfectly correlated between the sexes. Mutations with asymmetrical effects on each sex disproportionately contribute to total genetic variation for fitness and disease, relative to mutations with identical effects on both sexes. Given empirical estimates of the mutational distribution of fitness effects (Eyre-Walker and Keightley 2007), we also show that alleles with sex-differential effects should make a quantitatively large contribution to disease susceptibility within each sex. Indeed, under biologically plausible conditions, most of the total genetic variances for fitness or disease are attributable to alleles with strongly asymmetrical effects between the sexes. Sexually antagonistic alleles will further exaggerate the evolution of sex-specific genetic architecture for fitness and disease and therefore amplify the patterns described here.

Our model provides an evolutionary theoretical framework to explain why mutations with sex-specific effects are readily detected within association studies (Ober et al. 2008) and provides a new twist on the ‘common disease, common variant’ hypothesis (Lander 1996) by predicting that common, disease-causing variants are more likely to have sex-limited or sexually antagonistic effects than variants that are similarly selected in both sexes. We therefore emphasize that the hunt for genetic disease markers needs to be fine-tuned to the reality that disease-predisposing mutations with the largest effects in a given sex – those that are identifiable using modern statistical genomics approaches – are likely to contribute negligibly to disease in the other sex. This sexually dimorphic genetic architecture is a direct consequence of sex-specific selection pressures. A strategy that actively focuses on sex-specific genetic effects is therefore preferable to one that merely controls for the effects of sex when estimating genome-wide significance.

### Two sexes, one genome

Understanding the genetic component of human disease is an important aim of contemporary genetics as it may provide keys to unlocking new methods of treatment or diagnosis (Lander and Schork 1994). Common human diseases may be caused by variants at a single locus (Mendelian disorders such as the inborn errors of metabolism, Garrod 1923) or by a more complex network of many loci, as seems to be the case for most polygenic diseases (e.g., asthma, diabetes, epilepsy, hypertension, schizophrenia). It is mutation that causes disease, as changes in gene dosage or in the complex arrangements of amino acids usually compromise protein functionality. But *context* is everything. The conditional nature of mutations is something well established in traditional genetics, where the severity of individual mutations may exhibit a huge phenotypic range that depends on the environments in which they are expressed (e.g., the availability of particular nutrients or exposure to environmental factors; Ober and Vercelli 2011). In some cases, mutations are not deleterious, or even neutral to selection, but instead may be beneficial; perhaps enabling the bearer to perform some biochemical reaction more efficiently, metabolize a new substrate, or invade a new ecological niche. Mutation, after all, is the basis of evolutionary adaptation.

That some mutations may be deleterious in one context but beneficial in another is especially interesting in species with separate sexes, as males and females can be thought of as two distinct contexts or ‘environments’ within which genes must function (Rice and Chippindale 2001). Thus, mutations may typically have sexually dimorphic consequences in their effects on individual traits or on fitness – that is, they are said to have sex-specific effects. Some alleles may have beneficial effects when expressed in one sex and deleterious effects when expressed in the other. In these cases, the alleles are said to be *sexually antagonistic*. Because sexually antagonistic alleles do not experience purifying selection continuously (each allele spends an equal proportion of evolutionary time within male and female genomic backgrounds), they are particularly likely to reach intermediate population frequencies (Rice 1984; Connallon and Clark 2012; Dean et al. 2012) and contribute disproportionately to phenotypic variance.

Milder forms of sex-specific selection can also generate sexual dimorphism for the genetic basis of disease. For example, mutations that are harmful to both sexes, but have asymmetric fitness effects per sex, will disproportionately influence phenotypic variability within the sex associated with the greater effect size. To see why, consider the following heuristic, which is based on an extension of the standard theory of fitness variation maintained at a balance between recurrent mutation and purifying selection (Mukai et al. 1974; Charlesworth 1987). Suppose that a specific deleterious allele reduces fitness in male and female carriers by amounts *s*_*m*_ and *s*_*f*_, respectively. Given a mutation rate to this allele of *u*, the equilibrium population frequency of the allele will be *q*_*eq*_ ≈ 2*u*/(*s*_*m*_ + *s*_*f*_) (Hansen and Price 1999; Whitlock and Agrawal 2009), and its contribution to fitness variance within each sex will be ∼2*q*_*eq*_*s*_*m*_^2^ and ∼2*q*_*eq*_*s*_*f*_^2^, in males and females, respectively. When the allele's effect is completely symmetric (*s *= *s*_*m*_ = *s*_*f*_), its contribution to variation will be the same in both sexes, *that is,* ∼2*us* (Mukai et al. 1974; Hansen and Price 1999). When its effect is highly asymmetric (e.g., *s *= *s*_*m*_ ≫ *s*_*f*_), then its contribution to variation in the sex with larger effect will be ∼4*us*, which represents a doubling of effect relative to the symmetric case.

Within the context of disease genetics, mutations with sex-specific fitness effects – which include mutations with asymmetrical fitness effects between males and females, and mutations with sexually antagonistic effects (i.e., they are beneficial in one sex and harmful in the other) – may be responsible for elevated disease risk. Three lines of evidence suggest that sex-specific selection could be an informative perspective with which we can study human disease genetics. The first is that sexually antagonistic genetic variation appears to be common and has a large effect on overall fitness variance in several animal populations (Rice 1992; Chippindale et al. 2001; Foerster et al. 2007; Bonduriansky and Chenoweth 2009; Innocenti and Morrow 2010). Second, mutation accumulation experiments indicate that harmful mutations typically have asymmetric fitness costs between the sexes (Morrow et al. 2008; Mallet et al. 2011; Sharp and Agrawal 2013). Finally, genetic architecture for various traits, including a number of common human diseases, is often sex-specific (Mackay 2001; Ober et al. 2008). These observations indicate that selection operating on males and females may often be divergent and that the context within which a mutation exerts its phenotypic effect(s) is likely to differ between the sexes.

The considerations outlined above provide the basic biological intuition for predicting sex-specific genetic architecture for fitness-related phenotypes, including disease, yet we currently lack the necessary theory to quantify the likely affect of sex-specific selection on patterns of phenotypic variation. Several existing models characterize the importance of sex-specific fitness effects on the evolutionary dynamics and equilibrium conditions of genetic variation at individual loci (e.g., Kidwell et al. 1977; Nagylaki 1979; Rice 1984; Hansen and Price 1999; Patten and Haig 2009; Whitlock and Agrawal 2009; Fry 2010; Connallon and Clark 2012; Jordan and Charlesworth 2012; Mullon et al. 2012). However, this prior work has not considered how the distribution of mutant effects across loci, and within each sex, will affect the degree of differentiation between male and female genetic architectures for fitness and disease susceptibility. We therefore developed a simple population genetic model that characterizes the interaction between sex-specific selection among mutations and the genetic basis of fitness variability within each sex. We first explore how male and female fitness effects influence the equilibrium frequencies of individual alleles within a population and then use these results to characterize the relative contribution of asymmetrically selected alleles to the overall genetic variance in each sex. Implications of these findings are discussed with regard to the fields of human disease genetics.

## Model and results

A common assumption in the disease genetics literature is that disease manifestation statistically associates with decreased fitness, and therefore, that (i) disease susceptibility alleles are deleterious and (ii) alleles with more severe effects on disease susceptibility tend to be more deleterious to fitness than those with mild effects (Eyre-Walker 2010; Simons et al. 2013). Under these conditions, the genetic basis of many diseases will indirectly reflect genetic variation for fitness, and this view is central to our theoretical arguments.

Consider the following, idealized genetic model for disease predisposition in a population with separate sexes. Suppose that in the focal sex (hereafter, sex 1; the other sex will be called sex 2), the probability of developing a particular disease is influenced by the genotypes of *k* diploid loci within the genome. For simplicity assume that each of these loci has two allele types, a wild-type (‘normal’ allele, subscript *n*, at a population frequency of *P*) and a disease-predisposing allele (‘disease’, subscript *d*, at a population frequency of *q*), and that gametic disequilibrium between loci and allele frequency differences between the sexes are both negligible. These assumptions are reasonable given low mutation rates, per locus and small effects of individual alleles on fitness (as both seem reasonable; e.g., Eyre-Walker and Keightley 2007).

With respect to the *i*th of *k* loci (*i *= {1, 2,…, *k*}), the relative probability of progressing to the disease (scaled against the probability of disease in individuals homozygous for the wild-type allele, *A*_*n, i*_) follows:

**Table:** 

Genotype:	*A*_*n, i*_ *A*_*n, i*_	*A*_*d, i*_ *A*_*n, i*_	*A*_*d, i*_ *A*_*d, i*_
Disease susceptibility (sex 1):	0	*γ*_*i*_	2*γ*_*i*_

where *γ*_*i*_ represents the effect size of the disease allele at the *i*th locus. From these conditions, the contribution of the *i*th locus to population variability in disease predisposition is var(*i*) = 2*p*_*i*_*q*_*i*_*γ*_*i*_^2^, where *p*_*i*_ and *q*_*i*_ refer to the frequencies of *A*_*n, i*_ and *A*_*d, i*_ alleles, respectively. Assuming additive contributions to disease susceptibility by each locus, overall population genetic variability in disease predisposition is var(disease) = 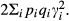 The contribution of the *i*th locus to overall disease predisposition in the population is 
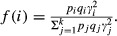


### Sex-specific fitness and equilibrium allele frequencies

Thus far, we have dealt with arbitrary allele frequencies and allelic effects. Yet these factors should be related, to the extent that disease progression lowers fitness (Eyre-Walker 2010; Simons et al. 2013). Natural selection influences the frequencies of disease-predisposing alleles by preferentially removing from the population mutations with relatively large deleterious effects. Assume that the fitness cost of carrying a disease allele is proportional to the degree to which it increases disease susceptibility. The relative fitness in sex 1, with respect to the *i*th locus, is as follows:

**Table:** 

Genotype:	*A*_*n, i*_ *A*_*n, i*_	*A*_*d, i*_ *A*_*n, i*_	*A*_*d, i*_ *A*_*d, i*_
Relative fitness in sex 1:	1	1−*s*_*i*_	1−2*s*_*i*_

where *s*_*i*_ = *cγ*_*i*_ > 0, and *c* is a positive constant relating disease to fitness (hence, the fitness ranking in the focal sex follows the order *A*_*n, i*_ *A*_*n, i*_ > *A*_*d, i*_ *A*_*n, i*_ > *A*_*d, i*_ *A*_*d, i*_). Given this proportionality, subsequent results can be expressed in term of fitness variation, without any loss of generality.

Because each locus spends an equal proportion of generations within male and female genomes, the evolutionary dynamics of disease-predisposing variants in sex 1 will also depend on the fitness effects of such variants in sex 2. For the *i*th locus relevant to sex 1, let the fitness parameterization for sex 2 follow:

**Table:** 

Genotype:	*A*_*n, i*_ *A*_*n, i*_	*A*_*d, i*_ *A*_*n, i*_	*A*_*d, i*_ *A*_*d, i*_
Relative fitness in sex 2:	1	1−*t*_*i*_	1−2*t*_*i*_

where *t*_*i*_ is the fitness effect of *A*_*d, i*_ on sex 2. Disease-causing alleles in sex 1 are deleterious to sex 2 when *t*_*i*_ > 0, neutral to sex 2 when *t*_*i*_ = 0, and beneficial to sex 2 when *t*_*i*_ < 0.

With small fitness effects per allele (2*s*_*i*_, 2*t*_*i*_ ≪ 1), the expected rate of allele frequency change per generation at the *i*th locus is well approximated by:


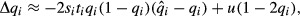
1

where *u* represents the rate of mutation at the locus, per gamete, and 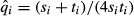 (see Connallon and Clark 2012; Mullon et al. 2012; Connallon and Clark 2013; Supporting Information). Selection maintains the disease allele indefinitely when 4*s*_*i*_*t*_*i*_ < *s*_*i*_ + *t*_*i*_ < 0. Under this scenario of balancing selection, and assuming weak mutation relative to the strength of selection, the population will evolve to a frequency near 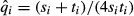. Otherwise, selection favors fixation of one of the alleles, and variation will be maintained at mutation-selection balance.

**Figure 1:**
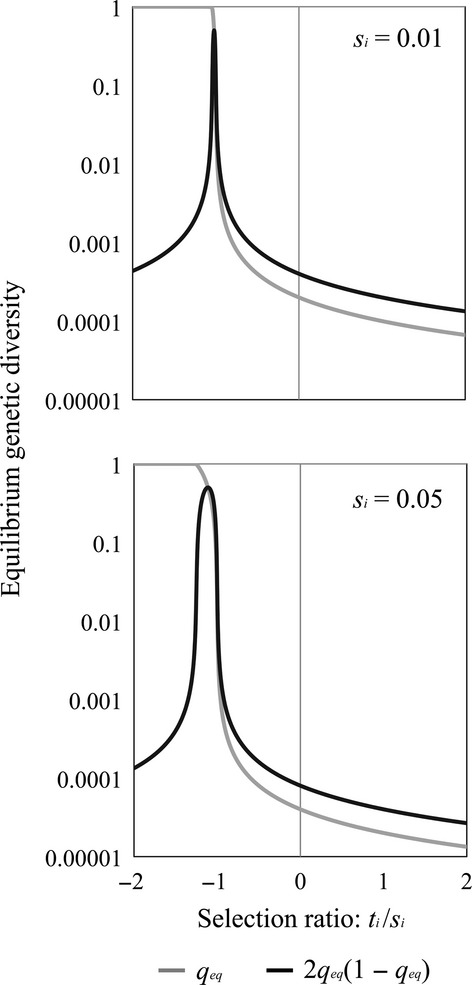
Equilibrium genetic diversity at a locus that harbors alleles with sex-specific fitness effects. Gray curves follow the frequency of an allele that is costly to sex 1 (each copy of the allele reduces fitness by amount *s*_*i*_; see the text for details). Black curves depict heterozygosity at the locus. The fitness effect on the other sex (sex 2) is also negative when *t*_*i*_ > 0 (i.e., *t*_*i*_/*s*_*i*_* *> 0). Its effect is positive, and the allele is sexually antagonistic, when *t*_*i*_ < 0 (i.e., *t*_*i*_/*s*_*i*_* *< 0). Results are based on numerical evaluation of the roots of Δ*q*_*i*_ = 0 [see eqn (1) and Supporting Information], with *u *= 10^−6^.

The equilibrium frequency (*q*_*eq*_) of the disease allele can be found by setting Δ*q*_*i*_ = 0, and finding the relevant roots (i.e., between zero and one; Supporting Information). Equilibrium allele frequencies and heterozygosity [*H *= 2*q*_*eq*_(*q*_*eq*_ – *q*_*eq*_)] are plotted in Fig. 1, which illustrates two important points about the population genetic consequences of sex-differential selection (for related results and discussion, see: Patten and Haig 2009; Fry 2010; Patten et al. 2010; Jordan and Charlesworth 2012; Connallon and Clark 2012; Mullon et al. 2012). First, across most of the parameter ranges of sex-specific selection, heterozygosity increases as *t*_*i*_/*s*_*i*_ decreases. Second, inflation of heterozygosity under sexual antagonism (*t*_*i*_/*s*_*i*_ < 0) is more pronounced with stronger selection at the locus (i.e., with increasing |*t*_*i*_| and *s*_*i*_). Because *s*_*i*_^2^*H* represents the relative contribution of a particular locus to fitness variance in sex 1, we expect that decreased purifying selection in sex 2 relative to sex 1 will elevate an allele's relative contribution to fitness variance in sex 1 (i.e., in the parameter range of *t*_*i*_/*s*_*i*_ < 1, which potentially includes sexually antagonistic selection).

### Contribution of asymmetrically selected loci to genetic variation for fitness and disease

To quantify the relative contributions of different allele categories to genetic variance for fitness or disease, we treat the sex-specific selection coefficients as random variables that vary among loci, with each locus contributing to the total variance within a given sex (we again focus on sex 1). The relative contribution of a random locus to disease is proportional to *q*_*eq*_(1−*q*_*eq*_)*s*^2^, where *q*_*eq*_ is a function of the mutation rate (assumed constant at *u*, per locus), and the sex-specific selection parameters, *s* and *t* (see above), which are treated below as random variables. We can consider how loci with sex-differential fitness effects contribute to variability in our focal sex (sex 1) by analyzing the relationship between *t*/*s* and the contribution of individual loci to the total fitness variance. With respect to the set of *k* loci, when the covariance between *q*_*eq*_(1−*q*_*eq*_)*s*^2^ and *t*/*s* is negative, alleles under weak purifying selection or positive selection in sex 2 will contribute disproportionately to disease in sex 1. We can formally define this hypothesis as cov[*q*_*eq*_(1−*q*_*eq*_)*s*^2^, *t*/*s*] < 0, which gives the criteria for disproportionately high sex-specific and sexually antagonistic effects on the disease phenotype of sex 1.

The parameters *s* and *t* will follow a bivariate probability distribution, and although this distribution is unknown (we return to this issue immediately below), we can define criteria for cov[*q*_*eq*_(1−*q*_*eq*_)*s*^2^, *t*/*s*] < 0 within the limit of small selection coefficient variance across the set of contributing loci. Suppose that *s* and *t* follow a joint probability distribution with correlation coefficient *r*_*st*_, and with marginal mean and variance: E(*s*), E(*t*), var(*s*), and var(*t*). By expanding *q*_*eq*_(1−*q*_*eq*_)*s*^2^ and *t*/*s* about the points *s *= E(*s*) and *t *= E(*t*), using the Taylor series, and assuming that most loci are within the parameter region of *t*/*s *> −1, then conditions for cov[*q*_*eq*_(1−*q*_*eq*_)*s*^2^, *t*/*s*] < 0 arise when:


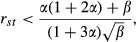
2

(see Supporting Information), where *α *= E(*t*)/E(*s*) and *β *= var(*t*)/var(*s*). When the marginal distributions are the same in each sex (E(*t*)/E(*s*) = var(*t*)/var(*s*) = 1), the criterion simplifies to *r*_*st*_ < 1. Thus, as long as allelic effects are imperfectly correlated between the sexes, then alleles that are more costly to sex 1 than sex 2 will disproportionately contribute to the disease phenotype in sex 1.

Estimates of the fitness effect distribution among spontaneous mutations suggest that the vast majority of functionally relevant mutations reduce fitness, and that the distribution of deleterious mutational effects is highly skewed, with most mutations having very small fitness effects (Eyre-Walker and Keightley 2007). Based on this body of research, we modeled the distribution of *t* and *s* using a bivariate gamma distribution and calculated the equilibrium contributions of individual loci to total additive genetic variance for fitness. Under this distribution, specific values of *s* and *t* are constrained to be positive, so that mutations are assumed to be deleterious to both sexes, and each locus evolves to equilibrium at mutation-selection balance, with the disease allele at a minor frequency. Because this eliminates the possibility of sexual antagonism, subsequent results establish a conservative, lower-baseline contribution of the loci that we are particularly concerned with (i.e., those where *t *< *s*), to phenotypic variance. For the set of loci that potentially contribute to the disease phenotype, suppose we are interested in the contribution of loci within some arbitrary parameter range (*a *< *t*/*s *< *b*) to the total genetic variability for the disease. Under an additive model, this contribution is 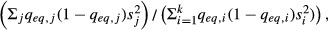 where *j* includes the set of loci meeting the criteria *a* < *t*/*s* < *b*.

**Figure 2:**
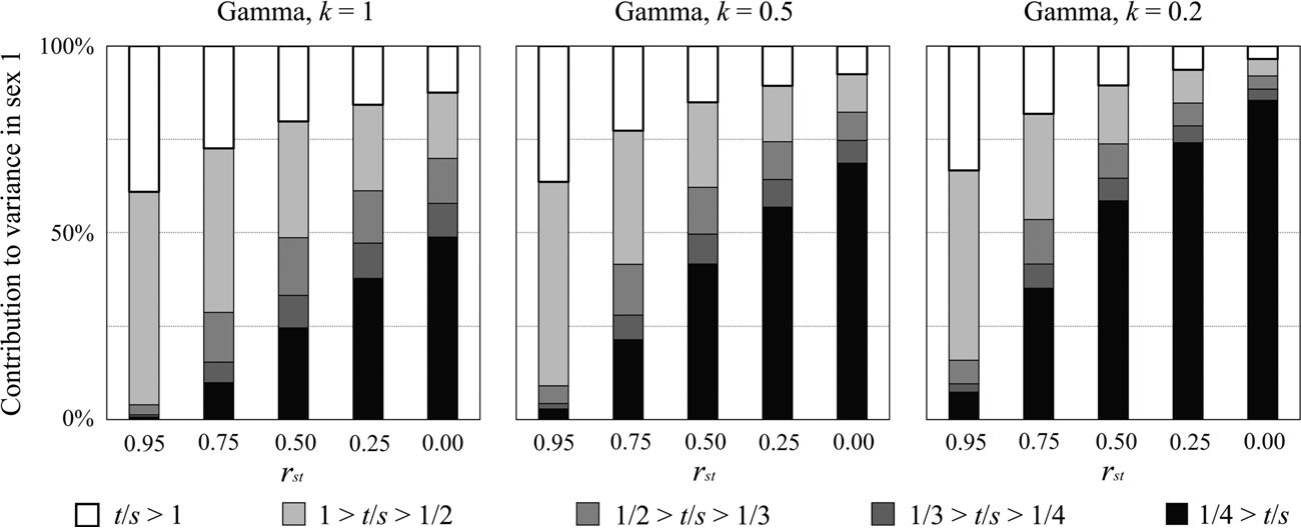
Relative contributions of asymmetrically selected alleles to sex-specific fitness variance. The term *t*/*s* represents the degree of asymmetry in selection between the sexes, with *t*/*s *=* *1 representing completely symmetric effects. Each column shows the relative contribution of specified allele classes (*a *< *t*/*s *< *b*) to the fitness variance in sex 1. The parameter space *t*/*s *<* *1 reflects the range of interest, where a deleterious allele in our focal sex (sex 1, which suffers a fitness cost of *s*) is less costly to fitness in the other sex (sex 2). Results are based on simulated data sets (1 000 000 mutations randomly sampled per column), with selection parameters drawn from a bivariate gamma distribution with equal marginals (shape and scale parameters, *k* and *θ*, with E(*s*) = E(*t*) = *kθ* = 0.02), and between-sex correlation of *r*_*st*_ (see Supporting Information for details).

We drew simulated parameter sets from a bivariate gamma distribution with equal marginal distributions for *s* and *t* (such that half of the loci have parameter sets with *t *< *s* and half have *t *> *s*) and calculated the equilibrium contributions of individual loci to the additive genetic variance in sex 1 (see Supporting Information). We focused on cases where the distribution of fitness effects is leptokurtic, as previously inferred from nonsynonymous polymorphism data from Drosophila and humans (Eyre-Walker and Keightley 2007; Keightley and Eyre-Walker 2007; Boyko et al. 2008). From these simulations, we find that, although only fifty percent of loci fall within the range *t *< *s*, such loci account for a large majority of the variance [see Fig. 2, which validates the general prediction of eqn (2), above]. This disproportionate contribution of asymmetrically selected loci to the total variance becomes more pronounced as the distribution of mutant selection coefficients becomes increasingly leptokurtic (i.e., as the gamma shape parameter, *k*, decreases below one and beyond). Mutations with at least a twofold stronger effect in sex 1, relative to sex 2, account for a majority of the fitness variance in sex 1, despite a strong positive correlation of selection coefficients between the sexes. For example, with a shape parameter of *k *=* *0.2 (as previously estimated from the sex-averaged fitness effect distribution of nonsynonymous mutations in humans; Keightley and Eyre-Walker 2007; Boyko et al. 2008), and between-sex correlation of *r*_*st*_ = 0.75, 82% of the variance in sex 1 is attributable to alleles with at least a twofold greater effect in sex 1 (*t*/*s *<* *1/2); 35% of the variance is attributable to alleles with a four-fold greater effect in sex 1 (*t*/*s *<* *1/4). These patterns become even more exaggerated with decreasing correlation of mutational effects between the sexes.

Each of the above results indicate that, as long as the effects of individual mutations are imperfectly correlated between the sexes – a condition supported by abundant evidence (see discussion, below) – patterns of genetic variation, including variation underlying disease phenotypes, are likely to be dominated by alleles with asymmetric fitness effects between the sexes. The overall contribution of such alleles to phenotypic variation is particularly high when the fitness effect distribution of deleterious mutations is leptokurtic (e.g., gamma with small shape parameter, *k *<* *1), which appears likely (Eyre-Walker and Keightley 2007).

## Discussion

We have presented a simple population genetic model of how alleles with sex-specific and sexually antagonistic effects can reach higher frequencies and explain a greater proportion of population-wide phenotypic variation in disease predisposition than alleles subject to equal purifying selection in both sexes. This result is intuitive for the case of sexual antagonism, as those alleles do not experience a continuous pressure of purifying selection across generations but instead are intermittently favored by selection. For mutations that increase disease predisposition in both sexes, the imbalance in selection pressures can greatly increase the effect of the allele within the sex that experiences a greater fitness cost of carrying the allele. Our model therefore predicts that alleles with asymmetric fitness effects per sex (e.g., sex-limited or sexually antagonistic) will account for a large proportion of genetic variance in disease susceptibility, even when such alleles arise rarely by mutation. Their inflated contribution to disease is driven by natural selection, which preferentially removes alleles with similarly strong effects in both sexes.

Our model obviously oversimplifies the potentially complex genetic basis of individual disease phenotypes, yet the qualitative results emerging from the model are nevertheless subject to relatively minor caveats. We assume throughout that the effects of individual alleles are additive (within and between loci). To the extent that population genetic variation is maintained at mutation-selection balance (rather than balancing selection), with small total mutation rate to disease-predisposing alleles, our neglect of dominance and epistasis will not greatly influence the results (e.g., Charlesworth 1987; in such cases, we can replace *s* and *t* with *sh* and *th*, with the latter representing the heterozygous effects of carrying a given disease allele; the scaling of parameters *th*/*sh* = *t*/*s* will of course remain the same). We also assume autosomal inheritance for all loci and thereby exclude contributions of X-linked variation. A longstanding prediction of sexual conflict theory is that sex chromosomes may be enriched for sexually antagonistic alleles maintained by balancing selection (Rice 1984; Patten and Haig 2009; but see, Pamilo 1979; Fry 2010; Connallon and Clark 2012; Mullon et al. 2012; Jordan and Charlesworth 2012), which is consistent with some lines of empirical evidence (Gibson et al. 2002; Innocenti and Morrow 2010; Pischedda and Chippindale 2006). Increased opportunities for balancing selection should elevate the contribution of sexually antagonistic alleles to phenotypic variance and could exaggerate the patterns predicted by our model. In addition, differences in ploidy between males and females, for each X-linked gene, can generate sex asymmetries in the phenotypic effects of segregating mutations, and further decouple the genetic basis of male and female fitness (James 1973; Cowley and Atchley 1988; Long and Rice 2007; Wayne et al. 2007; Connallon 2010). Overall, violation of our key assumptions should further exaggerate the degree to which the genetic basis of disease phenotypes, or fitness in general, is sexually dimorphic.

An important implication of this model is that a greater proportion of the heritable variation in fitness-related traits, such as disease, could be explained if sex-specific effects were taken into account in data analysis. However, this strategy has so far been largely overlooked (Maher 2008; Ober et al. 2008; Manolio et al. 2009; Magi et al. 2010). For pragmatic reasons, many genome-wide association studies either ignore gender by simply pooling samples of males and females, or male and female data sets are analyzed separately and then pooled if no sex-specific effects are found. Polymorphisms with major effects have been their main focus, and while alleles with sex-limited or strong sex-specific effects may be revealed using this workflow (Liu et al. 2012), it is unlikely that alleles with sexually antagonistic effects will, as they would need to achieve genome-wide levels of significance in both data sets. Furthermore, analyzing data in stratified and then pooled groups will increase the type I error rate due to the additional number of tests and is therefore an unattractive choice. An alternative is to incorporate sex and its interaction with genotype in the statistical model (e.g., using PLINK, Purcell et al. 2007). Although the power to detect an interaction effect is lower than the overall effect, unless its effect size is much greater (Brookes et al. 2004), this full-factorial approach is still more powerful than stratified or subgroup analyses (Behrens et al. 2011), and it is the approach we advocate here.

The model and its conceptual background do not allow us to make predictions about the specific diseases caused by alleles with sex-specific or even sexually antagonistic effects. Nevertheless, the important requirements of our model – an imperfect correlation of mutational effects between males and females, and a polygenic basis to trait variance – are general properties of quantitative traits (Mackay 2001; Poissant et al. 2010), which render our predictions broadly applicable. There is now widespread evidence for sex-by-genotype effects on many complex traits, including some common diseases (reviewed in Ober et al. 2008). A number of recent genome-wide association studies have also identified loci with significant male- or female-specific effects for autism spectrum disorder (Lu and Cantor 2012), coronary artery disease (Liu et al. 2012), types I and II diabetes (Consortium 2012; Orozco et al. 2012), schizophrenia (Shifman et al. 2008; Zhang et al. 2011), and Crohn's disease (Liu et al. 2012). However, despite clear genome-wide evidence of sexually antagonistic genetic variation being found in several model organisms, including humans (Stearns et al. 2012), very few putative sexually antagonistic loci have been reported in the literature. One example is a polymorphism in the promoter of monoamine oxidase A (*MAOA-VNTR*), where alleles vary in the number of repeats present (Sabol et al. 1998), a short variant increased risk of delinquent behavior in boys, whereas the girls with at least one copy of the longer variant had higher risk of delinquency (Åslund et al. 2011) – the effects however were dependent upon self-reported environmental factors during development (physical, sexual, and emotional abuse). This locus therefore appears, in some environments at least, to be experiencing conflicting (i.e., antagonistic) selection pressures in the two sexes. In other taxa, the putative examples reported again do not consistently exhibit opposing-fitness effects across the sexes (Smith et al. 2011; Khila et al. 2012). A comprehensive analysis of sexually antagonistic fitness effects at the level of the individual loci is however still missing.

Although genomic conflicts between the sexes have been implicated before in contributing to disease risk in humans (Frank and Crespi 2011), our model highlights explicitly the role that this recently emerged paradigm of evolutionary biology (Tregenza et al. 2006) may have in human disease genetics. It does not necessarily rely on cross-generational effects (such as imprinting), epistatic interactions between chromosomes, or deletions that reveal pathologies (Frank and Crespi 2011), and as such it should have general and widespread relevance to many diseases and conditions. This hypothesis could also help explain the apparent paradox of why several disease-causing variants are experiencing positive selection (Corona et al. 2010; Wu et al. 2012). The current favored explanation is that these alleles may have experienced positive selection in the past (perhaps because they were protective against ancestral pathogens), but they are now mismatched to modern environments. However, if these alleles are sexually antagonistic, then they may experience a net positive selective pressure but still cause disease in sex-specific manner. Note that these hypotheses are not mutually exclusive and due to the polygenic nature of many diseases, many processes may be at work simultaneously.

In summary, we have established a quantitative empirical framework for illustrating how disease predisposition alleles with asymmetrical or sexually antagonistic effects can be maintained in a population and disproportionately contribute to fitness variation. Our model is a new application of evolutionary principles to disease genetics by uniting processes of sex-differential selection to the field of Darwinian medicine. It makes clear predictions about the features of disease-causing alleles in terms of their effect size or equilibrium frequency. Importantly, they will only likely be revealed empirically if appropriate statistical models are applied. Specifically, the inclusion of sex-by-genotype interactions, to date something not normally included in genome-wide association studies. A secondary implication of this study is that if alleles with sex-specific or sexually antagonistic effects are responsible to some degree for contributing to disease risk, then longer-term therapeutic aspirations (summarized as ‘personalized medicine’) will also need to take gender into account.
