# Supplementary material for: Implications of sex-specific selection for the genetic basis of disease
Source: Evol Appl. 2013 Sep 4;6(8):1208–17. doi: 10.1111/eva.12097 (PMC3901550; doi:10.1111/eva.12097)
Supplement: Supplementary file 1 — Data S1 Supplementary material. [file eva0006-1208-sd1.docx]

**SUPPLEMENTARY MATERIAL**

**I. Allele frequency dynamics under mutation and sex-specific selection**

Following Connallon and Clark (2012, 2013), we assume the order of events, birth, selection, mutation, and random mating with respect to the locus in question. Assuming weak forces of mutation and selection at single loci, we can approximate the total change in the frequency of a disease predisposing allele (for sex 1) as:

Δ*q* = Δ*q_sel_* + Δ*q_mut_*

where Δ*q_sel_* is the change due to selection and Δ*q_mut_* is the change due to mutation. Assuming equal forward and backward mutation rates between alleles (*i.e.*, the mutation rate per allele copy is *u*, regardless of the identity of the allele), then Δ*q_mut_* = *uq* + *u*(1 – *q*) = *u*(1 – 2*q*).

Let *q*_1_ be the frequency of the *A_d_* in the gametes of sex 1, and *q*_2_ be the frequency of the *A_d_* in gametes of sex 2. Because each sex makes an identical genetic contributions to the next generation, the frequency change due to selection is given by Δ*q_sel_* = (Δ*q*_1_ + Δ*q*_2_)/2, where:

Let *q* = (*q*_1_ + *q*_2_)/2, *q*_1_ = *q* + *ε*, *q*_2_ = *q* – *ε*, where 2*ε* represents the allele frequency difference between breeding adults of each sex. Terms of *ε* will be on the order of the strength of selection, so that when selection is weak (*s*, *t* << 1; Nagylaki 1979; Charlesworth and Charlesworth 2010, p. 97), we can accurately approximate Δ*q* to first order in *ε*:

where $\hat{q}=(s+t)/(4st)$. The denominator is near unity under weak selection, leading to .

We solve for the equilibrium allele frequency, under mutation and selection, using Newton’s method, and iterating the following recursion to convergence, with starting frequency *x*_0_:

$$x_{t+1}=x_{t}-\frac{2stx_{t}(1-x_{t})(\hat{q}-x_{t})-u(1-2x_{t})}{2st[(\hat{q}-x_{t})(1-2x_{t})-x_{t}(1-x_{t})]+2u}$$

**II. Covariance between effect size and selection asymmetry between the sexes**

To determine conditions leading to cov[*q_eq_*(1 – *q_eq_*)*s*^2^, *t*/*s*] < 0, we assume that *u* << *s* + *t*, (valid when mutations meet the criteria, *t*/*s* > -1, and *u* is sufficiently small), for which we can approximate effect size as ~2*s*^2^*q_eq_*. Letting *f*(*s*, *t*) = *s*^2^/(*s* + *t*) and *g*(*s*, *t*) = *t*/*s*, leads to the revised condition, cov[*f*(*s*, *t*), *g*(*s*, *t*)] < 0. A Taylor series expansion of the left side of the inequality leads to:

$$\begin{matrix} \mathrm{cov}[f(s, t), g(s, t)]\approx\mathrm{var}(s)\left(\frac{\partial f(s, t)}{\partial s}\cdot\frac{\partial g(s, t)}{\partial s}\right)_{\begin{matrix} s=E(s) \\ t=E(t) \end{matrix}}+\mathrm{var}(t)\left(\frac{\partial f(s, t)}{\partial t}\cdot\frac{\partial g(s, t)}{\partial t}\right)_{\begin{matrix} s=E(s) \\ t=E(t) \end{matrix}} \\ +\mathrm{cov}(s, t)\left[\left(\frac{\partial f(s, t)}{\partial s}\cdot\frac{\partial g(s, t)}{\partial t}\right)_{\begin{matrix} s=E(s) \\ t=E(t) \end{matrix}}+\left(\frac{\partial f(s, t)}{\partial t}\cdot\frac{\partial g(s, t)}{\partial s}\right)_{\begin{matrix} s=E(s) \\ t=E(t) \end{matrix}}\right] \end{matrix}$$

which can be used to obtain eq. (2) of the main text.

**III. Simulation Procedure for data in Figure 2**

We simulated selection coefficients for random mutations using a bivariate gamma distribution, based on an algorithm proposed by Michael and Schucany (2002). For each mutation, we first sampled a value for *t* from a gamma distribution with shape and scale parameters, *k* and *θ*, where E(*t*) = *kθ* and var(*t*) = *kθ*^2^. Given *r_st_* < 1, values of *s* were then obtained by drawing from a gamma distribution with the following parameters (conditional on *t*):

*k*|*t* = *k* + *x*, where *x* ~ Poisson[*λ* = *tr_st_*/(*θ*(1 – *r_st_*))]

and

*θ*|*k* = *θ*(1 – *r_st_*)

Since *s* and *t* are both positive, by definition, and assuming weak mutation (*s* + *t* >> *u*), the contribution of a random locus to the fitness variance will be roughly proportional to ~*s*^2^/(*s* + *t*). For each distribution of selection coefficients, we simulated 1,000,000 random mutations and calculated the cumulative contribution of mutations within each specified selection category (*a* < *t*/*s* < *b*) to variance in sex 1.

**IV. Additional Reference**

Michael JR, Schucany WR. 2002. The mixture approach for simulating bivariate distributions with specified correlations. *The American Statistician* 56:48-54.
